# Association between dyslexia and overweight/obesity among Chinese children: findings from a cross-sectional study

**DOI:** 10.3389/fped.2025.1600848

**Published:** 2025-07-16

**Authors:** Bing Zhu, Hong Fan, Min Wang, Kaiheng Zhu, Yanan Feng, Haoxue Wang, Qi Jiang, Zhen Xiang, Qianhui Chen, Ziyan Xiong, Ranran Song

**Affiliations:** ^1^ Zhejiang Provincial Center for Disease Control and Prevention, Hangzhou, China; ^2^ Department of Maternal and Child Health and MOE (Ministry of Education) Key Lab of Environment and Health, School of Public Health, Tongji Medical College, Huazhong University of Science and Technology, Wuhan, China; ^3^ Quzhou Center for Disease Control and Prevention, Quzhou, China; ^4^ School of Life Sciences, Peking University, Beijing, China

**Keywords:** overweight, obesity, dyslexia, Reading disabilities, children

## Abstract

**Background:**

Extensive studies have consistently reported associations between neurodevelopmental disorders and overweight/obesity. However, limited research addressed the weight status of children with dyslexia. Therefore, this study aimed to explore the association between dyslexia and overweight/obesity among Chinese children.

**Methods:**

7,116 children were recruited from the Tongji Reading Environment and Dyslexia research program, including 197 dyslexic and 6,919 non-dyslexic children. The standard “Screening for overweight and obesity among school-aged children and adolescents (WS-T 586-2018)” was utilized to define overweight and obesity. Logistic regression models were employed to examine the association between dyslexia and overweight/obesity.

**Results:**

Among dyslexic children, 18.8% were overweight and 23.9% were obese. Among non-dyslexic children, 17.3% were overweight and 15.4% were obese. The proportion of obesity among dyslexic children was significantly higher than that among non-dyslexic children (*P* = 0.001). After adjusting for all potential confounders, children with dyslexia were more likely to be obese (OR = 1.57, 95% CI: 1.10-2.24). Analyses stratified by sex indicated that such associations were more significant among girls. No significant association was found between dyslexia and overweight.

**Conclusions:**

This cross-sectional study provides evidence that dyslexic children had significantly higher odds of obesity. This finding emphasizes the urgent need for heightened awareness and strategies targeted to mitigate excess body weight for this special population.

## 1 Introduction

Dyslexia is a common neurodevelopmental disorder characterized by impaired fluent or accurate word recognition and spelling, despite the individual having adequate intelligence and typical hearing and vision (1). The estimated prevalence of dyslexia ranges from 5% to 17.5% in English-speaking countries (2) and approximately 3.45% among Chinese school-aged children (3). As the most common type of learning disorder, dyslexia is associated with poor long-term academic achievements and professional development, resulting in costly burdens on individuals and society (4).

Previous studies have emphasized that children with neurodevelopmental disorders are at an increased risk of developing excess body weight. For example, Michael et al. discovered that children with attention-deficit/hyperactivity disorder (ADHD) had a 1.9-fold increased risk of being overweight and obese (5). Another study revealed a noteworthy disparity in the prevalence of overweight and obesity among autistic children aged 2–17 years compared to the general population in the United States (6). Children with intellectual disability (ID) also exhibited significantly higher odds of being obese compared to their healthy peers (7). The potential mechanisms explaining this significant association between neurodevelopmental disorders and excess body weight included unhealthy eating problems, inadequate physical activity, excessive sedentary behaviours, and antipsychotic medication taking (8–10). Individuals with autism spectrum disorder (ASD) may also exhibit conditions associated with obesity, such as sleep and gastrointestinal problems (11–13). Additionally, shared genetic liability might be another contributor to the significant association between neurodevelopmental disorders and obesity (14).

Notably, children with dyslexia are also exposed to risk factors associated with obesity, such as psychosocial stress, reduced physical activity, and maladaptive coping behaviours (15–17). However, there is a relative dearth of studies that explore the weight status among dyslexic individuals. To be best of our knowledge, only one study conducted in Iran has indicated a higher proportion of overweight and obesity among dyslexic children compared to their healthy counterparts (18). This study exclusively encompassed male students and relied on basic comparisons of proportions between the two groups. Previous studies have emphasized the importance of paying special attention to children with developmental disabilities when assessing the risk of childhood obesity (19, 20). Given the association between dyslexia and obesity-related factors, it is necessary to examine whether children with dyslexia are at an increased risk of excess body weight. Such insights could provide empirical evidence to develop targeted interventions, which may help to improve long-term health outcomes in children with dyslexia and reduce the societal and healthcare burdens associated with both conditions.

Based on the knowledge gaps above, this study utilized participants from the Tongji Reading Environment and Dyslexia (READ) program and aimed to investigate the association between dyslexia and overweight and obesity. We also explored possible sex-related differences in this potential relationship. To the best of our knowledge, this is the first study to examine the weight status of dyslexic children in China.

## 2 Method

### 2.1 Study population

**Figure 1:**
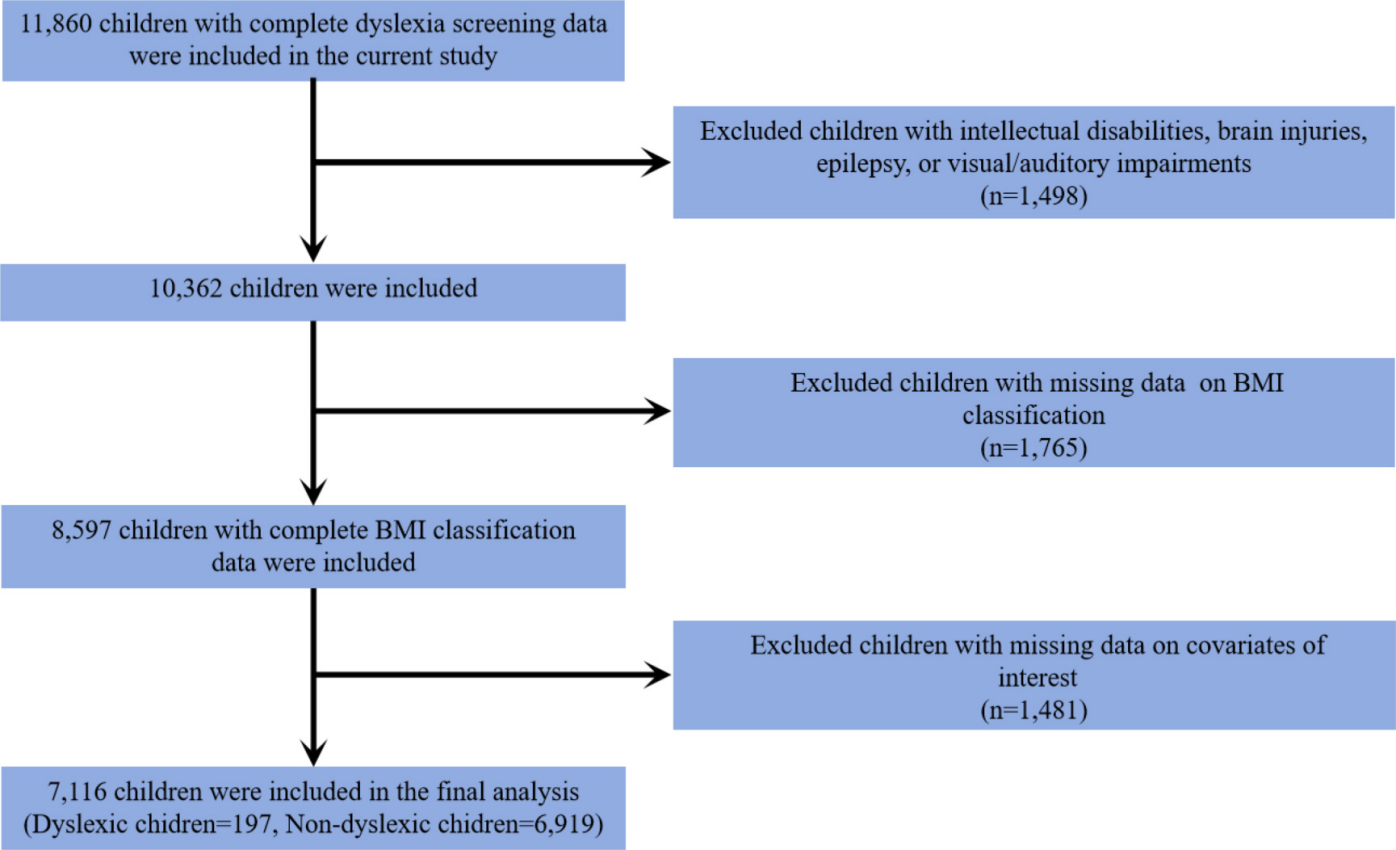
Flowchart of the study population included in the final analysis (*n* = 7,116).

This study included a subset of participants from the READ program, which has been described in our previous study (21). Initially, 11,860 children with complete dyslexia screening data were included. We further excluded children with intellectual disability, brain injury, epilepsy, and visual and auditory impairment (*n* = 1,498). In addition, children with missing data on body mass index (BMI) classification (*n* = 1,765) and covariates of interest (*n* = 1,481) were also excluded from this study. Ultimately, 7,116 eligible children were included, comprising 197 dyslexic children and 6,919 non-dyslexic children. The inclusion process of the study participants is presented in Figure 1.

This study was approved by the Ethical Committee of Tongji Medical College, Huazhong University of Science and Technology. Written informed consent was obtained from all participants.

### 2.2 Measurements

#### 2.2.1 Dyslexic screening

In this study, the dyslexic children were screened with the Dyslexia Checklist for Chinese Children (DCCC) and the Pupil Rating Scale-Revised Screening for Learning Disabilities (PRS). The DCCC, originally developed in 2006 and revised in 2018, was constructed based on the definition of dyslexia in the International Classification of Diseases (ICD-10), the Diagnostic and Statistical Manual of Mental Disorders (DSM-IV), and clinical symptoms described in the research literature (22). It is a parent-report questionnaire designed to assess the reading ability of children in grades 2–6. The DCCC consists of 57 items, each scored from 0 (never) to 5 (always), with higher total scores indicating poorer reading ability. The PRS has 24 items, which are filled by the header teacher, a higher score is associated with a better learning ability (23). Children with dyslexia were defined based on the following criteria: (a) the DCCC score was two standards higher than the average score for the same grade; (b) the score of the PRS was below 65; (c) the Chinese exam test score was in the bottom 10% of the class. (d) children without intellectual disability, brain injury, epilepsy, and visual and auditory impairment. These screening criteria have been systematically applied to investigate the prevalence and risk factors of dyslexia in the Chinese mainland (2, 3, 24).

#### 2.2.2 Weight status

Children's height and weight were measured through parental reports, and the BMI was calculated based on the following equation: BMI = weight (kg)/height (m^2^). Criteria for determining overweight and obesity were established according to age- and sex-specific BMI cut-off points outlined in the Screening for Overweight and Obesity Among School-Aged Children and Adolescents (WS-T 586-2018) guidelines (25). This standard is universally applied to school-aged children and adolescents aged 6–18 years across all regions of China.

#### 2.2.3 Covariates

Demographic characteristics, including children's age, sex, parental education level, and family annual income, were considered covariates in this study. Additionally, perinatal factors previously identified to influence children's body weight were taken into account as covariates in this study. These perinatal factors encompassed birth weight and gestational age. Birth weight was categorized into three groups: low birth weight (<2,500 g), normal birth weight (2,500–4,000 g), and macrosomia (≥4,000 g) (26). Likewise, gestational age was classified into three categories: 28–37 weeks, 37–42 weeks, and ≥42 weeks (27). The physical activity level was also introduced as a confounder in this study. Physical activity was measured utilizing the Physical Activity Rating Scale-3 (PARS-3). The PARS-3 questionnaire consists of three items, each with a score ranging from one to five, comprising intensity, duration, and frequency of the physical activity. The calculation of the total physical activity score is based on the following equation: intensity score × (duration score-1) × frequency score. The intensity of physical activity was categorized into three levels based on the total score: light (score ≤19), medium (score 20–42), and high (score ≥43) (28).

### 2.3 Statistical analysis

Categorical variables were presented as frequency (n) and percentage (%), and group differences were assessed using the chi-square test. Continuous variables were described with mean ± SD (standard deviation), and comparisons between groups were conducted with the Student's *t*-test. We employed multiple logistic regression models to estimate the odds ratios (OR) and corresponding 95% confidence intervals (95% CI) for the association between dyslexia and overweight/overweight. Two models were established in this study: Model 1 was a crude model without any adjustments, and Model 2 was adjusted for child age, sex, maternal educational level, parental education level, family annual income, child birth weight, gestational age, and the intensity of physical activity. To determine whether the association between dyslexia and overweight or obesity differs between boys and girls, we further conducted stratified analyses by sex.

All statistical analyses were conducted using SPSS statistical software (version 26.0) and a two-sided *P* < 0.05 was considered statistically significant for all analyses.

## 3 Results

### 3.1 General characteristics of the study population

The average age of non-dyslexic children was 10.27 (±1.49) years, and the average age of dyslexic children was 10.19 (±1.54) years. Compared to the control group, dyslexic children exhibited a significantly higher proportion of boys, accounting for 68.0% of the dyslexic group and 49.4% of the controls (*P* < 0.001). The maternal educational level was found to be lower for children with dyslexia (*P* = 0.017). There were no significant differences between the two groups concerning children's age, paternal educational level, family annual income, birth weight, and gestational age. In addition, it was observed that 15.7% of dyslexic children participated in medium to high levels of physical activity, a proportion significantly lower than that in non-dyslexic children (25.9%) (*P* = 0.001). The demographic characteristics of the study population are presented in Table 1.

**Table 1:** General characteristics of the study population.

Characteristics	Total (*n* = 7,116)	No-dyslexia (*n* = 6,919)	Dyslexia (*n* = 197)	*P* value
Age, years, mean (SD)	10.27 (1.49)	10.27 (1.49)	10.19 (1.54)	0.390
Grade				0.801
2	1,165 (16.4)	1,131 ((16.4)	34 (17.3)	
3	1,291 (18.1)	1,254 (18.1)	37 (18.8)	
4	1,408 (19.8)	1,366 (19.7)	42 (21.3)	
5	1,599 (22.5)	1,562 (22.6)	37 (18.8)	
6	1,653 (23.2)	1,606 (23.2)	47 (23.8)	
Sex, *n* (%)				<0.001
Boys	3,554 (49.9)	3,420 (49.4)	134 (68.0)	
Girls	3,562 (50.1)	3,499 (50.6)	63 (32.0)	
Maternal education level, *n* (%)				0.017
High school or below	4,631 (65.1)	4,487 (64.9)	144 (73.1)	
Beyond high school	2,485 (34.9)	2,432 (35.1)	53 (26.9)	
Paternal education level, *n* (%)				0.092
High school or below	4,694 (66.0)	4,553 (65.8)	141 (71.6)	
Beyond high school	2,422 (34.0)	2,366 (34.2)	56 (28.4)	
Family annual income, *n* (%)				0.479
<100,000 CNY/year	4,599 (64.6)	4,467 (64.6)	132 (67.0)	
≥100,000 CNY/ year	2,517 (35.4)	2,452 (35.4)	65 (33.0)	
Birth weight, *n* (%)				0.676
Low (<2,500 g)	817 (11.5)	794 (11.5)	23 (11.7)	
Normal (2,500–4,000 g)	5,549 (78.0)	5,392 (77.9)	157 (79.7)	
Macrosomia (≥4,000 g)	750 (10.5)	733 (10.6)	17 (8.6)	
Gestational age, *n* (%)				0.580
28–37 weeks	387 (5.4)	375 (5.4)	12 (6.1)	
37–42 weeks	6,266 (88.1)	6,097 (88.1)	169 (85.8)	
≥42 weeks	463 (6.5)	447 (6.5)	16 (8.1)	
Intensity of physical activity, *n* (%)				0.001
Light	5,292 (74.4)	5,126 (74.1)	166 (84.3)	
Medium to high	1,824 (25.6)	1,793 (25.9)	31 (15.7)	

SD, standard deviation.

### 3.2 Weight status of the study population

**Figure 2:**
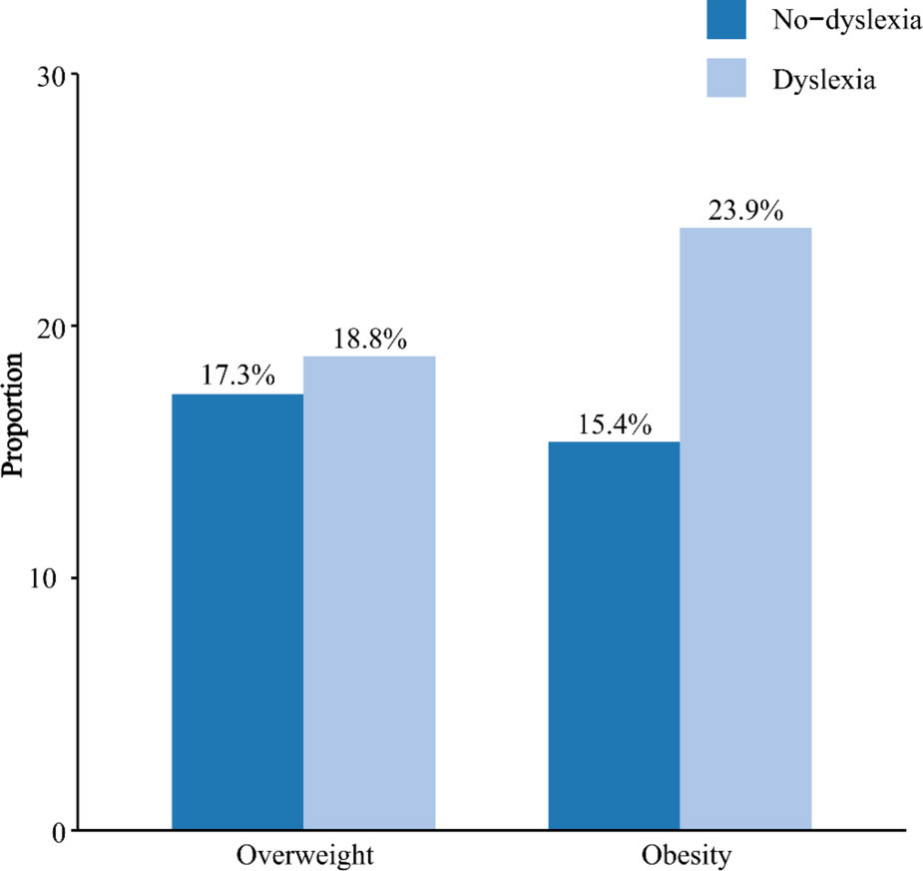
The proportion of overweight and obesity in children with dyslexia versus those without.

The weight status of the study participants was shown in Table 2 and Figure 2. The mean BMI of dyslexic children was 19.07 kg/m^2^, which was significantly higher than that of the control group (18.25 kg/m^2^) (*P* = 0.007). In the dyslexic group, the proportion of overweight was 18.8%, while in the control group, it was 17.3%. The proportion of obesity was found to be 23.9% in the dyslexic group and 15.4% in the control group. There were no statistically significant group differences between the dyslexic and the control group in terms of the proportion of overweight (*P* = 0.581). Compared to the control group, children in the dyslexic group had a significantly higher proportion of obesity (*P* = 0.001).

**Table 2:** Weight status of the study population.

Characteristics	Total (*n* = 7,116)	No-dyslexia (*n* = 6,919)	Dyslexia (*n* = 197)	*P* value
BMI, kg/m^2^, M (SD)	18.27 (3.42)	18.25 (3.40)	19.07 (3.91)	0.007
Overweight, *n* (%)	1,232 (17.3)	1,195 (17.3)	37 (18.8)	0.581
Obesity, *n* (%)	1,115 (15.7)	1,068 (15.4)	47 (23.9)	0.001

BMI, body mass index; M, mean; SD, standard deviation.

### 3.3 Association between dyslexia and overweight/obesity

Table 3 presents the results of the multivariable logistic regression analysis examining the association between dyslexia and overweight/obesity. In Model 1 (unadjusted), children with dyslexia had significantly higher odds of obesity compared to their non-dyslexic peers (OR = 1.81, 95% CI: 1.28–2.56). After adjusting for all potential confounding factors, the association remained statistically significant, with dyslexic children exhibiting 1.57 times the odds of obesity (OR = 1.57, 95% CI: 1.10–2.24). However, no significant association was observed between dyslexia and overweight in either the crude or adjusted models.

**Table 3:** The logistic regression model of the association between dyslexia and overweight and obesity.

Models	Overweight		Obesity	
	OR (95% CI)	*P* value	OR (95% CI)	*P* value
Model 1				
Dyslexia group				
No	1.00		1.00	
Yes	1.28 (0.88–1.86)	0.205	1.81 (1.28–2.56)	<0.001
Model 2				
Dyslexia group				
No	1.00		1.00	
Yes	1.10 (0.75–1.61)	0.638	1.57 (1.10–2.24)	0.013

Model 1 was a crude model without any adjustments; Model 2 was adjusted for child age, sex, maternal educational level, parental education level, family annual income, child birth weight, gestational age, and the intensity of physical activity.

Abbreviations: OR, odds ratio; CI, confidence interval.

Stratified analyses adjusted for potential confounders indicated that the association between dyslexia and obesity was significant among girls. Specifically, girls with dyslexia had 2.59 times the odds of obesity compared to non-dyslexic girls (OR = 2.59, 95% CI: 1.37–4.89). The association between dyslexia and obesity in boys did not reach statistical significance (Table 4).

**Table 4:** The logistic regression model of the association between dyslexia and overweight and obesity stratified by sex.

Sex	Overweight		Obesity	
	OR (95% CI)	*P* value	OR (95% CI)	*P* value
Boys				
Dyslexia group				
No	1.00		1.00	
Yes	0.89 (0.56–1.41)	0.616	1.33 (0.87–2.02)	0.193
Girls				
Dyslexia group				
No	1.00		1.00	
Yes	1.75 (0.90–3.39)	0.100	2.59 (1.37–4.89)	0.003

The stratified model was adjusted for child age, maternal educational level, parental education level, family annual income, child birth weight, gestational age, and the intensity of physical activity.

Abbreviations: OR, odds ratio; CI, confidence interval.

## 4 Discussion

This study provides valuable insights into the association between neurodevelopmental disorders and unhealthy weight conditions. In this study, it was found that around 20% of dyslexic children were overweight, and over 20% exhibited obesity. Dyslexic children were more likely to be obese compared to their non-dyslexic counterparts. However, no statistically significant association was observed between dyslexia and overweight. Sex-stratified analysis found that dyslexia was significantly associated with obesity in girls, but not in boys. This study, for the first time, investigated excess body weight among dyslexic children in China. It represents the initial endeavour to investigate weight-related issues in this specific population, contributing new insights to the existing knowledge.

This study demonstrated that dyslexic children engaged in fewer physical activities, which was consistent with the previous studies (16, 29). One possible reason was that children with dyslexia were more likely to experience motor problems (30, 31) and executive dysfunction (32). The motor problems may result in dyslexic children being less willing to engage in physical activities that require special motor skills, while the impaired executive function of dyslexic children makes it challenging for them to adhere to regular and sustained exercise routines. Besides, dyslexia can heighten various challenges for children, encompassing deficits in reading and math, difficulties in handling tasks, impairments of attention, as well as issues related to memory (33–35). These challenges may result in an increased amount of time spent on learning and homework for children with dyslexia, which in turn decreases the allocation of time for exercise.

Our findings revealed an increased odds of obesity among dyslexic children, aligning with previous studies that explored the association between various neurodevelopmental disorders and obesity. Several underlying mechanisms may contribute to this observed association between dyslexia and obesity. Firstly, insufficient physical activity and sedentary behaviours are robust risk factors for obesity, and the unhealthy lifestyle of children with dyslexia may contribute to their increased risk of obesity (36). Secondly, dyslexia is also associated with an increased risk of conditions linked to obesity, such as ADHD, depression, and anxiety disorders (37–39). These comorbidities may increase the risk of obesity in children with dyslexia. Finally, polymorphisms in genes such as *DRD2* and *BDNF* have been identified as shared factors associated with both dyslexia and obesity (40–44). These shared genetic factors may also play a role in the increased susceptibility to obesity among dyslexic children. More in-depth investigations are needed to further explore the underlying mechanisms between dyslexia and obesity.

When we further stratified the comparisons by children's sex, we observed that dyslexia was positively associated with obesity only in girls. This sex-specific association is consistent with findings from other neurodevelopmental disorders. For instance, a meta-analysis identified female sex as a potential risk factor for overweight and obesity in children with ASD (45), and another study found that ADHD was associated with obesity in adolescent girls but not in boys (46). Several mechanisms may explain this disparity. Compared to boys, girls with dyslexia may be more likely to cope with academic stress or low self-esteem through emotional eating, which may lead to excess weight gain (47). In addition, girls exhibited significantly lower levels of physical activity in this study, which may further contribute to their increased susceptibility to obesity when affected by dyslexia.

However, no significant relationship was found between dyslexia and overweight in this study. Lam and Yang also found that ADHD was linked to obesity but not overweight in Chinese adolescents (48). A meta-analysis comprising 15 studies revealed that individuals with ASD exhibited a higher prevalence of obesity compared to controls, while the prevalence of overweight did not differ significantly from that in the control group (49). In contrast, another study suggested a consistently elevated risk of overweight and obesity in individuals with ADHD (50). The reason for these inconsistent results remains unclear. The relationship between neurodevelopmental and excessive body weight is intricate, even bidirectional. Higher-quality studies are imperative to explore the correlations and causal relationships.

Some limitations were presented in this study. Firstly, because of the cross-sectional nature of the collected data, the causal relationship between dyslexia and obesity remains unclear. Secondly, children's height and weight were assessed based on parent-reported rather than objective measurements, which might result in some inaccuracies in measurements. However, evidence-based research has shown that parent-reported height and weight can provide a highly accurate classification of children's weight status, thereby supporting the reliability of our findings (51). Thirdly, the study population was restricted to specific regions and schools, which may limit the generalizability of the findings to broader populations. Finally, future considerations should encompass additional potential factors associated with obesity for children with dyslexia, such as eating patterns and sleep problems. Despite these limitations, this study provided new clues and considerations for future investigations, especially within the limited research context regarding the correlation between dyslexia and obesity.

## 5 Conclusion

In conclusion, this study provided evidence that children with dyslexia were at an increased risk of obesity, with this association was more significant among girls. Based on our findings, it is reasonable to implement weight screening and develop strategies for weight control among dyslexic children. Further high-quality studies are required to validate this association and delve into the underlying mechanisms of obesity in this special population.

## Data Availability

The datasets presented in this article are not readily available because to protect the privacy of participating parents and children, we did not make the data presented in this study publicly available. Requests to access the datasets should be directed to Ranran Song, songranran@hust.edu.cn.
